# Quality of Life and Role of Palliative and Supportive Care for Patients With Brain Metastases and Caregivers: A Review

**DOI:** 10.3389/fneur.2022.806344

**Published:** 2022-02-17

**Authors:** Adela Wu, Gabriela Ruiz Colón, Michael Lim

**Affiliations:** ^1^Department of Neurosurgery, Stanford Healthcare, Stanford, CA, United States; ^2^Department of Neurosurgery, Stanford University School of Medicine, Stanford, CA, United States

**Keywords:** brain metastases, palliative care, supportive care, quality of life, advanced cancer, caregiver

## Abstract

Brain metastases (BM) are the most commonly diagnosed secondary brain lesions in adults, influencing these patients' symptoms and treatment courses. With improvements in oncologic treatments, patients with BM are now living longer with their advanced cancers, and issues pertaining to quality of life become more pressing. The American Society of Clinical Oncology has recommended early implementation of palliative care for cancer patients, though incorporation and implementation of palliative and other supportive services in the setting of true multidisciplinary care requires additional attention and research for patients with intracranial metastases. We review the physical, cognitive, and psychosocial challenges patients with BM and their caregivers face during their cancer course as well as the current published research on quality of life metrics relating to this patient population and the diverse roles specialty palliative care, rehabilitation services, and other healthcare providers play in a comprehensive multidisciplinary care model.

## Introduction

Metastatic brain tumors are the most commonly diagnosed secondary brain lesions in adults, with an incidence of 8.3–14.3 per 100,000 people (1). Annually, ~150,000–200,000 people are diagnosed with brain metastases (BM) in the United States alone (2). Lung cancer, breast cancer, and melanoma are the primary malignancies most likely to predispose to development of BM, which may encompass metastatic leptomeningeal disease as well.

Typically, development of BM indicates advanced cancer, and patients may be frail and chronically ill by the time they present for additional surgery, chemotherapy or radiation. Furthermore, metastatic brain tumors can additionally impact patients' neurological and cognitive function and their overall quality of life. Often, BM management regimens already involve specialists from disparate disciplines, as new treatment options, such as immunotherapy, emerge and gain traction (3). BM patients therefore benefit from coordinated care from a multidisciplinary team, consisting of their oncologists, radiation oncologists, surgeons, as well as providers from palliative care, social work, and therapy services if necessary to address needs in a holistic manner.

Outcomes from early implementation of palliative care (PC) in particular have been investigated in several studies, including randomized controlled trials, notably Temel et al. (4). The landmark study determined that patients with metastatic non-small cell lung cancer benefited from early PC with improvements in survival and quality of life. However, most advanced cancer patients do not necessarily receive early PC referral, as 48% of a cohort of BM patients received PC consultation with median timing of consultation to death of 1.6 months (5).

We present a comprehensive review of not only the challenges patients living with metastatic brain tumors and their caregivers face but also validated measures of quality of life before delving into a discussion of critical palliative and supportive care providers and resources that may enhance quality of life.

## Challenges of Living with Brain Metastases

Patients with metastatic brain tumors face unique challenges due to their disease. The treatments for BM also present potential short-term and long-term complications. Depending on their location, metastatic brain tumors lead to variable clinical presentations even while the primary cancer may be quiescent. Common symptoms include headaches (40–50% of patient presentations), seizures (15–20% of patient presentations), as well as different neurologic deficits, such as motor or language deficits (6). However, while the range of neurologic and cognitive symptoms may be large among metastatic brain tumor patients, the development of BM typically portends poor overall prognosis. For example, median survival for renal cell carcinoma patients with BM was 5 months, and the median survival for patients with solid BM from non-small cell lung cancer was 8.4 months (7, 8).

Fortunately, with treatment advances, certain BM patients can achieve good tumor control burden. Almost half of patients diagnosed with BM have a single, isolated intracranial metastasis at presentation, and they may undergo a variety of effective treatments, including surgery and stereotactic radiosurgery (9). Depending on the treatment regimen selected, patients with isolated BM have median survival ranging from 28.9 to 62.8 months (10). Combination therapies confer benefits to quality of life for this patient population as well; 88% of those who underwent surgery and whole brain radiation therapy (WBRT) reported improvement in Karnofsky Performance Status (KPS) scores (10). Even patients presenting with multiple BM have viable treatment options. Surgery and radiosurgery both have comparable tumor control and survival outcomes for patients with 2–4 BM (11). Separately, a large-scale prospective study included patients with 1–10 newly diagnosed BM and found that stereotactic radiosurgery alone conferred similar survival benefit [HR 0.97, 95% CI 0.81–1.18 (less than non-inferiority margin), *p* = 0.78; *p* non-inferiority < 0.0001] and adverse event profiles for patients with a few BM or 5–10 BM (12). Repeat stereotactic radiosurgery can also lead to good metastatic brain tumor control without side effects of radiation necrosis (13). Furthermore, in addition to radiation therapy, developments in chemotherapy and oncologic immunotherapy have also been promising for BM patients with various primary cancers (14–16).

However, the treatments available—whether in the form of surgery, radiation, or chemotherapy—for patients once metastatic brain tumors have developed can also be taxing and present risks. Even though the BM patient population is heterogeneous, those presenting with multifocal BM or leptomeningeal disease may have additional challenges with their treatment as well.

Neurosurgical procedures inherently involve potential risks and complications following intracranial tumor resection, such as superficial or deep wound infection, perioperative stroke, or postoperative hematoma (17). In addition, patients who are preoperatively frail, according to an 11-factor modified Frailty Index, are significantly more likely to develop life-threatening complications or mortality in a population of benign meningioma patients who underwent cranial surgery (18). Similarly, in a cohort of 180 geriatric patients with surgically-resected BM, the frailest patients according to the modified Frailty Index had significantly shortened median overall survival compared to those considered “least frail” (3 vs. 18 months, *p* < 0.0001). Furthermore, not all cases of BM are amenable to surgery, particularly if the tumors are multifocal, located in eloquent areas or pose greater risk than benefit during surgical resection. In these cases, patients with BM could be eligible for other forms of treatment.

At times, patients must resort to radiation therapy or palliative radiation, even though some people's overall clinical response may be minimal (19). Common adverse effects from cranial radiation include headache, nausea, vomiting, and fatigue among others. While the overall toxicity from radiation courses, particularly with goal of palliation, is typically mild and rare, some patients may still experience various Grade 1 or 2 adverse effects, such as mucositis and skin reactions, as well as higher grade toxicities (20, 21). On the other hand, up to 90% of brain tumor patients who undergo radiation therapy experience cognitive changes, which may be exacerbated by the treatment length, radiation dose, fraction size, and volume treated (22–24). In other cases, whole brain radiation therapy (WBRT) may be indicated for palliation as well and is a standard therapy for patients with multiple BM (25). However, WBRT carries risk of new symptoms in the future for cancer patients. Memory loss and cognitive impairment have been reported for up to 50% of patients who had undergone WBRT with a higher rate of developing dementia in young cancer patients over time (26). Brown et al. designed a multi-institutional study on the cognitive effects of WBRT. They found that patients who received both WBRT and stereotactic radiation had significantly worse cognitive performance (decline in verbal fluency as well as both immediate and delayed memory tasks) at 3-month follow-up than patients who did not undergo WBRT (27). At present, hippocampus-avoiding WBRT is an option for patients with multiple BM as this treatment protocol minimizes hippocampal atrophy (28).

Various medications may be prescribed for symptomatic relief or prophylaxis for patients with BM. Some patients may present with or will be at risk of developing seizures, and prophylactic anti-epileptic drugs like levetiracetam or phenytoin may be administered to decrease this symptomatic burden (29). However, a meta-analysis did not find significant decrease in seizure occurrence with prophylactic anti-epileptic medication compared to control [OR = 0.939, 95% confidence interval (CI) = 0.609–1.448, z = 0.29, *p* = 0.775] (30). Patients with BM commonly take corticosteroids to alle*via*te symptoms arising from vasogenic edema surrounding some intracranial metastatic tumors. Unfortunately, steroids have numerous adverse effects, such as mood shifts, hyperglycemia, and weight gain, and does not have permanent therapeutic effects.

Chemotherapy regimens are updated once a cancer patient develops metastatic brain tumors, in part due to the need for surmounting the blood-brain barrier and other factors that influence therapeutic levels of medications intracranially (31). However, even cancer patients without metastatic brain tumors can develop cognitive deficits from systemic chemotherapy in both short and long term cases (32). Targeted cancer immunotherapy has been a superb option for patients with metastatic cancer. However, the majority of early clinical trials assessing targeted therapies for advanced cancer patients excluded those with BM. Currently, many more clinical trials enroll patients with BM with primary cancer diagnoses of melanoma, non-small cell lung cancer, and breast cancer (33). Adverse effects from immunotherapy, some of which can be severe and debilitating, should not be overlooked even though they may herald good clinical response to therapy. In a cohort of 56 patients with Stage IV melanoma, 36% of the group experienced any immune-related adverse events associated with their anti-programmed death 1 (anti-PD1) treatment (34). Every patient had an adverse event while on ipilimumab treatment. In a separate retrospective chart-based study, it appeared that patients with melanoma and BM had longer median intracranial progression-free survival when they experienced severe adverse events following immunotherapy compared to those without severe adverse events, though the effect was not statistically significant (19.9 vs. 10.5 months, *p* = 0.053) (35).

The potential for patients' psychological distress must not be overlooked while providing healthcare for patients with BM. For patients with advanced cancer with or without BM, they typically experience high levels of distress and anxiety. For instance, a cross-sectional pilot study involved metastatic non-small cell lung cancers who did and did not have intracranial metastatic spread; 53% of the group of 78 patients had BM (36). Both groups of patients reported death anxiety that was significantly associated with demoralization (*p* < 0.001) and illness intrusiveness (*p* = 0.001). Cordes et al. studied groups of breast cancer patients with and without metastatic brain tumors, evaluating for measures of distress, depression, and anxiety. For patients with BM who underwent cranial radiotherapy in this study compared to people without intracranial metastases, a large proportion of the group (70 vs. 66%) experienced distress and reported higher measures of distress (*p* = 0.029) (37).

With increasing numbers of patients with BM, more families and caregivers also experience various challenges and burden. From a pilot study involving 21 family caregivers of patients with BM, Ketcher et al. (38) found that caregivers devoted extensive time and energy to providing care but lacked adequate support for numerous psychosocial aspects, such as coping mechanisms, anxiety, and depression. And, in general, caregivers of patients with BM with greater caregiving burden are at greater risk of suffering from anxiety and depression (39). Indeed, lower levels of resiliency appeared to correlate with high caregiver burden (OR = 0.76), according to the eQuiPe study, a prospective, longitudinal observational study involving advanced cancer patients and their family caregivers (40). Furthermore, caregivers reporting high burden were also less informed about the importance of self-care (OR = 0.39), pointing toward potential avenues for intervention in future prospective studies on building resiliency, reducing burden, and providing support for caregivers.

## Quality of Life in Patients with Brain Metastases

### Measuring Quality of Life

The World Health Organization (WHO) defines health related quality of life (QoL) as “an individual's perception of their position in life in the context of the culture and value systems in which they live and in relation to their goals, expectations, standards, and concerns” (41). While the complexities of capturing all elements of this multidimensional definition have been previously discussed across medical specialties, present research in metastatic brain tumors has focused on using validated scales to quantify functional status, neurocognitive abilities, and social wellbeing (42–45). The use of these scales has allowed providers and researchers to measure QoL throughout the course of treatment, and has served as a tool to document improvements, stability, or deterioration in a patient. Specifically, three scales have been commonly used: (1) the Karnofsky Performance Status (KPS), (2) Functional Assessment of Cancer Therapy-Brain (FACT-Br), and (3) EuroQoL-5D (EQ-5D) (Table 1).

**Table 1:** Commonly used Quality of Life (QoL) scales in BM literature.

**Scale**	**Domains assessed**	**Scoring**
Karnofsky Performance Status	Functional status, as defined by ability to carry on normal activity and work, as well as additional assistance necessary	0% (death) to 100% (no evidence of disease, no symptoms)
		• Scores between 80–100% are in Category A (able to carry on normal activity and to work; no special care needed)
		• Scores between 50–70% are in Category B (unable to work; able to live at home and care for most personal needs; varying amount of assistance needed)
		• Scores in 0–40% are in Category C (unable to care for self; requires equivalent of institutional or hospital care; disease may be progressing rapidly).
Functional Assessment of Cancer Therapy: General (FACT-G) and Brain (FACT-Br)	Physical wellbeing, social/family wellbeing, relationship with doctor, emotional wellbeing, functional wellbeing, additional concerns (FACT-Br specific)	0 (poorest QoL) to 200 (best QoL)
		• While some items are reverse scored, in general, an item is given a score of 4 if it is “not true at all” (indicating best QoL outcome for patient), score of 3 if it is true “a little bit,” 2 if it is true “somewhat,” 1 if it is true “quite a bit,” and 0 if it is true “very much” (indicating worst QoL outcome for patient)
EuroQoL-5D	Mobility (walk), self-care (washing and dressing self), usual activities (work, study, housework, family or leisure activities), pain/ discomfort, and anxiety/ depression	11,111 (no problems in any of the domains) to 55,555 (severe problems/ inability to perform task in all five domains)
		• QoL is coded using a 1 if no problem, 2 if slight problems, 3 if moderate problems, 4 if severe problems, and 5 if unable to or have extreme problems for each of the five domains
Spitzer Quality of Life Index	Activity, daily living, health, support, outlook	0 (poorest QoL) to 10 (best QoL)
		• Each domain is given score of 0, 1, or 2
		• 0 for each domain corresponds to not being able to perform activity or ADL, being very ill, receiving poor support, and being seriously confused, frightened or anxious
		• 1 for each domain corresponds to conducting normal activities and ADLs with assistance, feeling low on energy, perceiving limited family/friend support, and feeling some anxiety
		• 2 for each domain corresponds to performing normal activities and ADLs independently, feeling well, feeling strong relationships with others, and appearing calm
European Organization for Research and Treatment of Cancer (EORTC) Quality of Life Questionnaire-Brain Neoplasm (QLQ-BN20)	Symptoms (headaches, seizures, drowsiness, hair loos, itchy skin, leg weakness, bladder control), future uncertainty, visual disorder, motor dysfunction, communication deficit	0 (best QoL) to 100 (worst QoL) • 20 items rated on a four-point Likert Scale (not at all, a little, quite a bit, very much) • Linearly transformed to a 0–100 scale
European Organization for Research and Treatment of Cancer (EORTC) Quality of Life Questionnaire (QLQ-C30)	Five functioning scales (physical, role, cognitive, emotional, and social), symptom scales (fatigue, pain, nausea/vomiting), single symptoms (dyspnea, appetite loss, sleep disturbance, constipation, diarrhea, financial impact) and global health status	• For functioning and global health scales: 0 is worst QoL, 100 is best QoL • For symptom scales: 0 is best QoL, 100 is worst QoL

The KPS was introduced to describe a patient's ability to carry their normal activity and work, and their ability to care for themselves (46). The scale places patients into one of three conditions (47):

- Able to carry on normal activity and work. No special care is needed.
- Unable to work. Able to live at home, care for most personal needs. A varying degree of assistance is needed.
- Unable to care for self. Requires equivalent of institutional or hospital care. Disease may be progressing rapidly.

One major limitation of the KPS is that it focuses on physical functioning and need for assistance but fails to isolate neurocognitive drivers of ability to work or care for self.

On the other hand, the FACT-Br is a tailored subscale used in conjunction with the FACT-G, the general scale. Together, they measure physical wellbeing (e.g., nausea, energy, pain), social and family wellbeing (e.g., emotional support, family communication), relationship with doctors, emotional wellbeing (e.g., worries about death), functional wellbeing (e.g., ability to work, sleep well), and additional neurologic-specific concerns (e.g., problems with vision or hearing, ability to read or write like they used to) (48, 49). A limitation of this scale is that it uses a Likert scale to assess the presence of symptoms in the past 7 days, thus, recency or recall biases might affect the scoring. Moreover, it may not be appropriate to track deterioration, stability, or progress over longer time frames.

Lastly, the EQ-5D asks patients to rate their abilities across five domains using a descriptive scale ranging from “I have no problems [with activity]” to “I am unable to do [activity].” The five domains in the scale are mobility (ability to walk), self-care (ability to wash or dress self), usual activities (including work, study, housework, family or leisure activities), pain/ discomfort, and anxiety/depression (50). Importantly, this scale attempts to capture anxiety and depression, which have been shown to occur in advanced disease, including stage IV cancers (37, 51). However, the scale's brevity may prevent providers and researchers to understand specific drivers of poorer QoL. Given the wide range of symptoms that may result from brain metastases, assessment of QoL should seek to address both the concerns outlined by the WHO, as well as the functional, emotional, and psychiatric changes that may result from tumor burden.

It should be noted that numerous other QoL scales have been validated and are widely used. Other commonly used scales in the BM literature are included in Table 1.

### Quality of Life in Brain Metastasis

BM without any intervention results in poor survival, and a rapid decline in QoL (52). However, the rise in availability of multiple treatment modalities for BM has led to a growing body of literature describing survival and QoL after intervention (Table 2). Most commonly, WBRT, Gamma Knife Surgery (GKS)/stereotactic surgery (SRS), and surgical resection, have been examined in observational and randomized controlled trials.

**Table 2:** Summary of literature describing quality of life in patients with BM receiving treatment and their caregivers.

**References**	**Design and sample size**	**QoL scale(s)**	**Findings**
Wong et al. (53)	Prospective, *n* = 217	KPS, QLQ-C30, QLQ-C15-PAL, QLQ-BN20, FACT-BR, Edmonton Symptom Assessment Scale, Spitzer Quality of Life	• In a 12-week study period, fatigue, drowsiness, and appetite deteriorated from baseline at statistically significant level
			• Appetite loss, weakness, and nausea significantly increased from baseline, while balance, headache, and anxiety decreased from baseline
			• At baseline all symptoms assessed (e.g., nausea, pain, insomnia, concentration) except for appetite loss were significantly correlated with overall QoL
Steinmann et al. (54)	Prospective, *n* = 46	QLQ-C30, QLQ-BN20, DEGRO-LQ	• Global QoL remained stable in 3-month study period
			• Overall physical functioning deteriorated in the 3-month study period at a statistically significant level.
			• There was a statistically significant deterioration in drowsiness, hair loss, and weakness but headaches and seizures improved
Doyle et al. (55)	Prospective, *n* = 60 patient/ caregiver pairs	FACT-BR	• In 2-month study period after WBRT, the physical wellbeing domain had the greatest absolute deterioration (statistically significant level)
Wong et al. (56)	Prospective, *n* = 129	Spitzer Quality of Life	• After WBRT, daily living, health, and headache improved in 12.2, 21.1, and 18.9% of patients, respectively
			• After WBRT, 56.7% of patients had worsened fatigue and 53.3% had poor neurofunctioning status
Caissie et al. (57)	Prospective, *n* = 108	QLQ-C15-PAL, QLQ-BN20	• Following WBRT, insomnia, future uncertainty, visual disorder, and concentration significantly improved
			• There was a decrease in physical function and increase in emotional functioning
Mulvenna et al. (58)	RCT, *n* = 538 (269 WBRT + OSC, 269 OSC alone)	EQ-5D	• There was no evidence of a difference in QoL between patients receiving WBRT + OSC and OSC alone
			• There is an increase in symptoms in patients after receiving WBRT (increased drowsiness, hair loss, nausea, and dry or itchy scalp)
DiBiase et al. (59)	Prospective, *n* = 20	Spitzer Quality of Life	• Extracranial tumor progression after GKRS is associated with worsened Spitzer QoL score, whereas in patients with stable or improved tumor control, Spitzer scores increased
Skeie et al. (60)	Prospective, *n* = 97	FACT-BR	• For 66% of patients, mean QoL score improved at 9 months after SRS, and remained unchanged for 6% of patients
			• Local control, improved symptoms, and reduced need for steroids after GKRS is associated with higher QoL
			• Low QoL is associated with local failure, increased need for steroids, and progression of the peripheral disease
Habets et al. (61)	Prospective, *n* = 97	QLQ-C30, QLQ-BN20	• Physical functioning and fatigue worsened at 6 months after SRT
			• KPS <90 and tumor volume > 12.6 cm^3^ were associated with lower QoL scores at 6 months after SRT
Verhaak et al. (62)	Cross-sectional, *n* = 92	FACT-BR, Hospital Anxiety and Depression Scale, Multidisciplinary Fatigue Inventory	• Compared to the general population and adult cancer patients, BM patients had lower QoL scores for emotional wellbeing and most (57.6%) of patients reported problems with emotional wellbeing
			• Compared to the general population, patients with BM had poorer functional wellbeing, and general QoL before treatment
			• Compared to the general population, BM patients had higher social wellbeing scores
Bragstad et al. (63)	Prospective, *n* = 44	FACT-BR	• 12 months after GKS, physical, social, emotional, and functional wellbeing average remained unchanged from baseline
			• Asymptomatic BMs at baseline, higher KPS score, and lower RPA classes were associated with higher QoL after GKS, whereas age, sex, number of BMs, prior treatment, SRS dose, extent of peritumoral edema, mutation status, and baseline metastases to other sites did not predict QoL
Salvati et al. (64)	Retrospective, *n* = 62 (32 multiple metastases, 30 with a single metastases)	KPS	• Preoperative KPS in patients with multiple metastases was 83.1 vs. 82.3 in patients with single metastases
Saria et al. (39)	Descriptive cross-sectional, *n* = 56 caregivers of patients with BM	NA	• Caregivers most commonly deployed the following coping strategies against cognitive dysfunction in their relatives: acceptance, planning, positive reinterpretation and growth
Papadakos et al. (65)	Cross-sectional, *n* = 109 patients with BM and 77 caregivers	NA	• The most important information patients and caregivers want belongs to the medical and physical health domains (e.g., symptoms, side effects, cognitive impairment)
			• Caregivers prefer one-on-one counseling for all informational domains, including medical, physical, emotional, social, and spiritual informational needs

#### Whole Brain Radiation Therapy

Historically, WBRT was considered a mainstay of treatment for BMs. Early studies in QoL in BM provided prospective, descriptive analyses of QoL after WBRT. For instance, in one study conducted by Wong et al. (53), 217 patients who received WBRT between 2005 and 2012 were prospectively assessed for progression of symptoms and QoL using the KPS and FACT-Br scales, among others. In this study, overall QoL scores deteriorated over the three-month study period, and fatigue, drowsiness, and appetite were shown to deteriorate from baseline at statistically significant levels (53). In the first month following WBRT, weakness and appetite loss were the two elements which increased in severity at statistically significant levels, whereas in the second month, five symptoms (nausea, balance, headache, anxiety, and appetite loss) declined most severely. In the third month, anxiety was statistically significantly different than baseline. Notably, the authors did not find a difference in symptoms in patients taking dexamethasone (80% of patients), except for insomnia in the first month (53). In another prospective study of 46 patients by Steinmann et al., self-assessed global QoL remained stable in the 3-month follow-up period, but physical function deteriorated significantly (54). In this same study, QoL assessment by healthcare proxies, though, was found to be statistically significant lower at 3 months vs. baseline, and symptoms of fatigue, nausea, pain, dyspnea, appetite loss, and constipation were found to deteriorate, with statistically significant differences in fatigue and appetite loss, consistent with the findings by Wong et al. (54). Doyle et al. (55) also noted the poor concordance between proxies' assessment of health and patients' own self-assessment, and similarly found a trend toward poorer QoL, as defined by the FACT-G and FACT-Br scales. Moreover, similar to Steinmann et al.'s work, physical wellbeing at 2 months was found to deteriorate the most. However, in another study of 129 patients receiving WBRT, daily living and health, two elements of Spitzer Quality of Life Index, were found to significantly improve after treatment, and frequency of headache and fatigue declined (56). The authors suggest that while overall QoL may have not meaningfully improved, WBRT may have contributed to the stabilization of some symptoms. Improvement in QoL elements after WBRT was also noted in a study of 108 patients undergoing WBRT by Caissie et al., where improvements in sleep disturbance (insomnia), visual disorders, communication deficits, and future uncertainty were noted to improve (57).

Beyond the aforementioned observational studies, the 2016 Quality of Life after Treatment for Brain Metastases (QUARTZ) trial provided further evidence by assigning 538 patients with NSCLC to WBRT and supportive care or supportive care alone (58). This trial failed to show a difference in survival and QoL between the two treatment groups. Patients receiving WBRT and supportive care had 46.4 days quality-adjusted life-years (QALYs) vs. 41.7 days in the supportive care group alone. While these QALYs suggest a 4.7-day advantage for the WBRT group, the 90% confidence interval of −12.7 to 3.3 does not allow for a definitive conclusion proving survival and QoL advantage in the WBRT group. Moreover, the prevalence of severe or moderate QoL impairments, as measured by EQ-5D, was similar in patients with WBRT vs. those with supportive care, and deterioration, as measured by the KPS, was similar in both groups (58). Literature assessing QoL in patients receiving WBRT alone since the QUARTZ trial has been limited, largely due to pivot toward radiosurgery in select patients (66, 67).

Moreover, the utility of WBRT as adjuvant therapy in SRS or surgical resection was studied through in the European Organization for Research and Treatment of Cancer phase III trial, a study with 359 patients (68). While adjuvant WBRT was found to reduce intracranial relapses and neurologic deaths, the time period with functional independence was not increased, suggesting decreased QoL despite better tumor control (68). These findings are consistent with a later study by Brown et al. demonstrating that in patients with one to three metastases, SRS alone—without adjuvant WBRT—leads to less cognitive deterioration at 3 months, which may contribute to better QoL (67).

#### Stereotactic Radiosurgery

Quality of life in stereotactic radiosurgery (SRS) treatment of BM has been studied as early as 2002, when DiBiase et al. reported QoL outcomes in 20 patients undergoing Gamma Knife Radiosurgery (GKRS) (59). Using the Spitzer QoL survey and KPS, the authors demonstrated that among the 40% of patients whose tumor progressed after GKRS, QoL decreased. On the other hand, patients whose tumor did not progress, QoL remained stable or improved at one, three, and 6 months after treatment (59). As one of the earliest studies examining QoL in SRS, this study demonstrated a relationship between tumor burden in QoL, and showed that GKRS treatment can contribute toward stable or improved QoL. These findings have been replicated with larger samples, including a 97-patient study by Skeie et al. (60) which utilized the KPS and FACT-Br scales. Patients who had improved symptoms after GKRS had FACT-Br scores that were 4.6 points higher than those who experienced clinical deterioration. Importantly, a decline in QoL was noted among patients who required dexamethasone at the time of GKRS, and separately, this study found no association between prior WBRT status and post-GKRS QoL. Two important conclusions can be drawn from this study: first, reducing steroid use in the setting of peritumoral edema may confer a QoL benefit, and second, while WBRT alone may lead to cognitive decline and negatively impact QoL, WBRT does not seem to be a risk factor for better or worse QoL after GKRS (60). Skeie's findings regarding corticosteroids were also corroborated in a study by Habets et al. (61) assessing 97 patients with BM, though findings were not found to be statistically significant. Interestingly, though, corticosteroids were not found to negatively influence results of neurocognitive functioning over time, a measure that is often tested alongside QoL (61). Moreover, Habets et al. were among the first to establish a baseline difference in QoL among patients in BM vs. healthy controls, showing meaningful differences in global health status, physical functioning, emotional functioning, role functioning, and cognitive functioning as assessed by the QLQ-C30 scale (61). Similar to other studies, Habets et al. found that patients with progressive disease after SRS had poorer QoL scores over time, whereas those without disease progression had stable or even improved QoL scores. Declines in QoL were driven by poorer physical functioning, fatigue, and motor dysfunction, as assessed by the BN20 scale (61). Lastly, neurocognitive functioning was stable up to 6 months after SRS in patients with up to three BMs. In another prospective study by Veerhak et al., QoL was assessed in 92 patients set to undergo SRS. QoL prior to SRS were evaluated using the KPS, FACT-Br, Multidimensional Fatigue Inventory, and Hospital Anxiety and Depression scales to identify baseline deficits. Overall, 64.1% of patients had a clinically meaningful low QoL score in at least one of the subscales prior to SRS (62). Specifically, patients with BM were found to have significantly lower emotional wellbeing when compared to both general adult population and adult cancer patients. Patients with BM, though, were also found to have higher levels of social wellbeing, which the authors posit may be due to increased support patients experience just before undergoing treatments, such as SRS (62). When considering psychiatric wellbeing, 42.4 and 32.6% of patients met criteria for at least mild symptoms of anxiety and depression, respectively (62).

While many of the studies on SRS are from heterogenous patient samples with multiple primary tumor types, one study by Bragstad et al. focused on lung cancer, only, the most common origin of BMs. In their work, the authors identified baseline predictors for improved or stable QoL after GKRS. Total BM volume ( ≤ 5 cm^3^ vs. >5 cm^3^) at baseline was the only predictor associated with improved QoL after GKRS, as measured by the FACT-Br scale (63). On the other hand, asymptomatic BMs at baseline, higher KPS at baseline, lower recursive partitioning analysis (RPA) class at baseline were all predictors of high and stable QoL after GKRS. Importantly, in this subset of patients, baseline number of BMs, prior treatment, GKRS dose to the cranium, peritumoral edema, and baseline metastases to bone, liver, adrenals, or lymph nodes did not affect QoL scores (63). Overall, the authors found that 77% of patients improved and 82% had stable or improved cerebral symptoms at their last follow-up, supporting the use of GKRS as the preferred treatment modality in lung cancer patients with brain metastases (63).

#### Surgical Resection

While surgical resection is commonly used in the treatment of BMs, non-review research exploring QoL after surgical resection is limited. A 32-patient series of patients with one to three metastases reported patients' KPS preoperatively and used it as a surgical prognostic factor. Among the patients in this study, those with either single or multiple BM had similar proportions of metastatic tumor type, with lung metastases being most common. Notably, this sample's average KPS of 83.1 would place the average patient in Category A, meaning they can carry on normal activity and work with no special care necessary (64). Such a high preoperative KPS appears to reflect surgical candidacy and patient selection on the neurosurgeons' part, as the inclusion criteria for this study involved KPS > 60, isolated or up to three metastatic intracranial lesions, and, notably, controlled primary disease. There was no postoperative KPS reported.

#### Caregivers

Beyond patients' experiences, caregivers' QoL ought to be understood. As integral members of patients' care teams, caregivers take on significant emotional, physical, and load throughout their relatives' course of care (69). Patients with BM, specifically, represent a patient cohort that has advanced disease which may portend a greater load than a non-BM cancer patient, as advanced disease may indicate longer length of disease, rapid deterioration, or a terminal status. A study by Garzo Saria explored BM caregivers' experience specifically by analyzing patient's cognitive impairment against their caregiver's resiliency and coping strategies (70). The authors found that increased memory problems had a significant negative correlation with caregiver resilience, and acceptance, planning, positive reinterpretation, active coping, and suppression of competing activities serving as the most common coping mechanisms (70). Thus, it is important to preserve resilience and support caregivers in developing their coping strategies. Another need of caregivers is the ability to remain well informed in the caring of their relative. In one study by Papadakos et al., caregivers and patients were surveyed to understand their needs. Caregivers and patients prioritized information related to physical and medical matters (e.g., side effects, symptoms, headache management, seizure management). They preferred to receive this information *via* one-on-one counseling and pamphlets (65).

#### Drivers of Declining Quality of Life

Collectively, the studies described above demonstrate five key drivers for declining quality of life in patients with BM and their caregivers. These drivers can be reframed as opportunities to enhance end of life care (Table 3):

- Support declining physical and motor functioning
- Promptly consult psychiatric and psychological support services for both patients and caregivers
- Encourage and foster social connection to preserve emotional wellbeing
- Frequently share information with caregivers, especially around physical (e.g., symptoms) and medical (e.g., prognosis) matters
- Carefully review medications to limit side effects
- A multidisciplinary team is required to meet these diverse needs for both patients and caregivers as patients elect to receive treatment or opt for comfort measures.

**Table 3:** Thematic analysis of the needs of patients with BM and their caregivers, as determined by drivers of poor QoL.

**Needs of patients with BM and their caregivers**	**References**
Support for declining physical and motor functioning, including services from physical therapists or physical medicine and rehabilitation physicians	(54–57, 61)
Early consultation of psychiatric and psychological support services for both the patient and caregivers	(39, 53, 61, 62, 71)
Ability to stay connected to social networks to preserve emotional wellbeing	(61, 69)
Frequent information sharing with caregivers, especially around expectations on physical and medical matters	(65, 69, 70)
Careful and frequent medication review to limit side effects, with special attention to dexamethasone	(53, 60, 61)

## Role of Multidisciplinary Care and Palliative/Supportive Care Services

Metastatic brain tumor patients and their families benefit from effective patient-provider communication as well as comprehensive multidisciplinary care for surveillance, treatment, and preservation of high quality of life. Several randomized control trials have shown the benefits of early palliative and supportive care involvement for patients with advanced cancers (4, 72). However, implementing palliative care and other supportive care services requires organization at the provider and clinic levels. Danielson and Fairchild (73) describe the Rapid Access Palliative Radiotherapy Program (RAPRP) for the metastatic brain tumor clinic, with overarching goals of coordinating timely consultations and treatment and multidisciplinary care. The interdisciplinary team consisted of members from radiation oncology, nursing, social work, occupational therapy, and dietary services. Eighty six percentage of patients involved in the 6-month pilot study reported high satisfaction, with 97% of patients willing to recommend the program to other patients. In preparing a high-quality multidisciplinary care center for patients with BM, additional integral aspects also involve palliative care specialists interfacing with the treatment team consisting of oncologists, radiation oncologists and surgeons as well as involving various key stakeholders from social work, rehabilitation and nutrition services, nursing, psychological services, and more given the unique profile of challenges patients with BM face.

### Specialty Palliative Care

The American Society of Clinical Oncology (ASCO) issued a provisional clinical opinion and recommendation for the timely introduction and integration of palliative care (PC), broadly defined as specialized care for patients with serious illnesses, into standard cancer care when the patient is diagnosed with metastatic cancer or high symptom burden (74). Temel et al. randomized and fully evaluated 107 non-small cell lung cancer patients with metastatic burden to either standard oncologic care or early PC integrated into standard care (4). The early PC patient group demonstrated improvements across quality of life measures (mean FACT-L score 98.0 for early PC vs. 91.5, *p* = 0.03), proportions of patients suffering from depressive symptoms (16% for early PC vs. 38%, *p* = 0.01), and median survival (11.6 months for early PC vs. 8.9, *p* = 0.02) (4). More recently, Temel et al. also ran a multi-institutional randomized trial focused on early PC for patients with advanced, incurable cancer. Due to missing data and significant morbidity among the enrolled patient population, no measures were ultimately found to be statistically significant (75).

Other studies have found overall poor adherence to the ASCO recommendation (5, 76). For example, McDermott et al. investigated that only 48% of non-small cell lung cancer patients with BM received PC consultation during their disease course, although timing of PC consultation and rate of PC consultation have increased in 2016–2018 compared to trends in 2012–2015 (5). Only 19% thoracic oncologists from a single-institution study referred their patients with advanced lung cancer to PC specialty care. A separate nationwide database study found that metastatic non-small cell lung cancer patients did benefit from lower healthcare costs following specialty palliative care usage (77). Furthermore, oncology providers may have differing opinions about the breadth, meaning, and usage of PC, as evident from responses gleaned from semi-structured interviews conducted with oncology clinical trial investigators, researchers, nurses, and physicians (78).

ASCO recommends referral to interdisciplinary specialty PC for patients with advanced cancer (79). The ENABLE II study divided patients between advanced practice nursing PC and usual care, finding improved depression symptoms and QoL measures for the intervention group (80). Similarly, another cluster randomized trial demonstrated increased QoL at 4-month intervention follow-up for Stage III and IV patients enrolled in early PC at an independent PC clinic compared to standard care (81). However, additional studies, including randomized controlled trials, for specifically patients with BM are needed for insight on the role of specialty PC in comprehensive cancer care.

### Social Work

Social workers may perform a variety of roles when caring for patients with intracranial metastases. With broad training in counseling, care coordination, community resource management, and other patient-centered skills, social workers are uniquely positioned to provide a number of critical services for cancer patients and families. Meier and Beresford argue that social workers in palliative care, for instance, have the specific knowledge and skillset to advocate and give psychosocial support for patients as well as facilitate care (82). In planning for end of life, social workers provide key communication skills while conducting advance care planning for patients, as they have more experience and expertise discussing advance directives than nurses or physicians (83). However, social workers, even those with additional training devoted to palliative care, face challenges in defining their roles within the multidisciplinary team: “social workers in palliative care need to make themselves heard [and] visible and conduct joint visits. …I get more buy-in after other team members watch me work,” states Higgins who is a social work on a palliative care team at Brigham and Women's Hospital/Dana-Farber Cancer Center (82). Currently, relatively little is known about the role of social work in care for patients with BM, and the field would benefit from future research and attention to the important role social workers play.

### Physical Therapy and Other Rehabilitation Services

A critical aspect of post-surgical and therapeutic recovery and maintenance of functional fortitude involves utilization of rehabilitation services for patients with BM. Not only do brain tumors themselves provoke various neurologic and systemic symptoms but the treatment courses patients undergo once BM develop is physically taxing and fraught with adapting to different potential deficits. For example, steroids are commonly prescribed for brain tumor patients to control manifestations of vasogenic swelling, but side effects include fatigue, muscle wasting, and weight changes (84). Rehabilitation services broadly involve the expertise of physical medicine and rehabilitation providers, physical and occupational therapists as well as speech and language therapists. Occupational therapists engage patients in exercises to overcome barriers that negatively impact an individual's physical, social, and emotional needs. Physical therapists work with patients to improve their strength, flexibility, balance and fine motor movements. Speech and language therapists perform various evaluations for speech, cognitive, language and swallowing abilities in addition to teaching patients exercises to improve their language and cognitive function.

Over 80% of patients with central nervous system tumors require rehabilitation services (85). In a separate study, Mukand et al. found that most brain tumor patients suffered from cognitive deficits (80%) and motor deficits (78%), with 39% of the cohort describing five or more separate neurologic deficits (86). A separate survey of 25 brain tumor patients revealed that 84% of the group reported recent fatigue, with worse symptoms experienced by those with recurrent lesions (87). However, following rehabilitation, the patients reported improvements across several functional scales, including KPS, Modified Barthel Index, and Motricity (motor function) Index. Similarly, another study of ten primary brain tumor patients indicated that total functional outcome significantly improved across three functional measures post-rehabilitation with a delayed enhancement in quality of life 1 month following discharge (88). Outcomes from inpatient rehabilitation are not significantly disparate between benign and malignant brain tumors or primary and secondary intracranial lesions, although more research is required for specifically metastatic brain tumor patients (89). Tang et al. included patients with BM as well as glioblastoma and other brain tumors who underwent inpatient rehabilitation, and patients demonstrated improved functional scores compared to measures on admission with a significant correlation between high functional improvement and longer survival (90). As for evaluating outpatient rehabilitation, there are several potential indices, such as the Functional Assessment of Cancer Therapy, to identify brain tumor patients who could potentially benefit from rehabilitation services (48). A group based in Canada sought to understand the population of brain tumor patients who received occupational therapy by examining demographics of 3,199 patients, of which 78.2% had malignant lesions (91). A recent randomized controlled trial enrolled functionally independent glioma patients currently on treatment who either underwent standard rehabilitation care or a supervised rehabilitation course (92). The specialized rehabilitation course involved 6 weeks of physical therapy with a focus on cardiovascular and resistance training, evaluations of patients' progress and performance with activities of daily living, and individually tailored exercises when appropriate. The intervention group exhibited superior aerobic strength (β = 2.6), cognitive functioning (β = 16.2), and decreased fatigue (β = −13.4).

Cognitive support and rehabilitation services are an especially important aspect of holistic care for patients with BM as well. Cognitive dysfunction may manifest as impairments in memory, language, and executive function, which can impact decision-making capacity for treatment and personal decisions (93). The vast majority of brain tumor patients (80%) experience cognitive deficits depending on tumor location, size, and grade (86). There are some preventative methods to protect cognitive ability when patients are faced with treatment choices. Hippocampus-avoiding WBRT significantly curtails the risk of developing memory loss, and proton radiation therapy involves lower entrance and exit doses that can spare brain tissue and preserve cognition (94). Some providers may also consider prescribing neuroprotective agents, such as memantine and renin-angiotensin-aldosterone system blockers (95). Cognitive rehabilitation harnesses principles of neuroplasticity in retraining or promoting compensation training for brain tumor patients. Such rehabilitation exercises benefit patients most when implemented early, such as a study demonstrating that postoperative brain tumor patients regained some cognitive function just after a few weeks (96, 97).

Overall, research on multidisciplinary efforts to promote quality rehabilitation programs for brain tumor patients is still lacking. A particular challenge for rehabilitation specialists lies in the heterogeneity of needs within this patient population, since therapy programs are ideally personalized based on clinical status and needs (98). Such a premise necessitates open and timely communication among various members of the oncologic multidisciplinary team. A review of randomized and non-randomized clinical trials found one low-quality controlled clinical trial encompassing 106 glioma patients, some of whom were enrolled in an individualized, outpatient multidisciplinary rehabilitation program, involving occupational, social, psychological, and physical therapies (99). Despite high overall drop-out rate (20% at 6 month follow-up), patients in the specialized therapy group had improvements in self-care, mobility, locomotion, communication as well as cognition (*p* < 0.05 for all) at 3-month follow-up (99). As with social work's role in the interdisciplinary care team for BM patients, rehabilitation services also lack a firm place in most oncology practices, even though they can provide essential aid for patients at any stage in their cancer course (98).

### Additional Gaps in Multidisciplinary Care

Patients with BM have unique perspectives on their prognoses and describe various needs. A qualitative study by Dorman and Pease involved collecting semi-structured interviews of nine patients with intracranial metastases from non-small cell lung cancer (100). Several patients expressed the importance of prioritizing quality of life along with preserving mobility and cognitive function. In particular, numerous studies have recapitulated the particular emotional and psychological distress cancer patients and patients with brain tumors experience. Personalized psychosocial support for patients with BM can lead to significant improvements in measures of distress, anxiety, and depression, as evident from a pilot study of 59 primary malignant brain tumor patients who worked with a certified psycho-oncologist. However, other patients with BM may indicate that they do not require additional help and, thus, reject services (101). Barriers to appropriate supportive care continue to exist and prevent some patients from accessing and utilizing services, pointing to a need for addressing patient knowledge and awareness.

### Supporting Caregivers

Over time, awareness of the caregiver experience for patients with advanced cancers has increased. At present, caregiver burden—the multifaceted experience and reaction to patient needs and demands—is relatively well-studied in cancer research (102, 103). Among the unique burdens faced by caregivers of patients with BM is the extensive longitudinal cancer caregiving experience as patients with intracranial metastases are living longer with improved treatments. Furthermore, as the number of patients with metastatic brain tumors rises due to longer survival, the amount of caregivers will similarly increase, pointing to the importance of more research in this area of caregiving.

Caregiver wellbeing is a potentially fruitful aspect for investigation as well as for implementation of support services. Ketcher et al. collected self-reported information about caregiving responsibilities and wellbeing from 21 family caregivers of patients with BM. Overall, the study participants reported moderate levels of caregiver burden, which was itself significantly associated with time spent on caregiving (R = 0.59, *p* < 0.01), anxiety levels (R = 0.54, *p* < 0.05), depression levels (R = 0.59, *p* < 0.01), and efficacy of coping (R = −0.54, *p* < 0.05) (38). One small pilot study investigated outcomes after implementing a program that involved two 90-min in-person sessions at the patients and caregivers' homes and one 30-min telephone appointment (104). Trained oncology nurses facilitated the sessions with patient-caregiver dyads. Results demonstrated significantly improved measures of quality of life for caregivers (t = 2.992, *p* < 0.006), while the patients' emotional wellbeing trended toward a statistically significant improvement.

Overall, patient and caregiver communication and coordination with their healthcare providers remain critical throughout the cancer course. While one study of 600 stage IV cancer patients and 346 caregivers demonstrated that patients found communication with physicians to be well-executed compared to caregivers' opinions, both groups reported worse perceptions of physician communication and care coordination when anxious (105). Dionne-Odom et al. examined outcomes following implementation of a clinic-based telemedicine support system (FamilyStrong) for caregivers of patients with grade IV brain tumors (106). A palliative care nurse interfaced with caregivers on a weekly basis, evaluating for distress and advocating for various support services, including local counseling services and coordinating with the primary neuro-oncology team for patient care needs. However, overall there are few published studies specifically including and targeting caregivers of patients with BM.

## Conclusion

As brain tumor and cancer treatments improve, patients with brain metastases (BM) have longer survival, though they still face numerous physical and psychosocial challenges from their disease and therapies. Patients with BM would benefit from coordinated multidisciplinary care consisting not only of their oncologists and surgeons but also among palliative care specialists, rehabilitation therapists, nursing, and other key healthcare providers. There is a dearth of published literature focused on quality of life studies, illness experiences, and the role of palliative and supportive care for this particular patient and caregiver population. This review highlights the important and gaps in understanding aspects of high-quality multidisciplinary care for patients with BM.

## Author Contributions

AW and GC contributed to draft manuscript preparation. AW, GC, and ML reviewed the manuscript. All authors designed the general outline for the manuscript. All authors contributed to the article and approved the submitted version.

## Funding

AW was supported by an individual fellowship from Agency for Healthcare Research and Quality (1F32HS028747-01).

## Conflict of Interest

ML has research support from Arbor, BMS, Accuray, Tocagen, Biohaven, Kyrin-Kyowa, and Urogen. The remaining authors declare that the research was conducted in the absence of any commercial or financial relationships that could be construed as a potential conflict of interest. The handling editor declared a past co-authorship with one of the author ML.

## Publisher's Note

All claims expressed in this article are solely those of the authors and do not necessarily represent those of their affiliated organizations, or those of the publisher, the editors and the reviewers. Any product that may be evaluated in this article, or claim that may be made by its manufacturer, is not guaranteed or endorsed by the publisher.
